# Solitary Fibrous Tumour: A Rare Differential Diagnosis of Unilateral Nasal Mass

**DOI:** 10.7759/cureus.36901

**Published:** 2023-03-30

**Authors:** Zi Hao Chew, Eng Haw Lim, Mohd Eksan Sairin, Aneeza Khairiyah Wan Hamizan

**Affiliations:** 1 Department of Otorhinolaryngology Head & Neck Surgery, Universiti Kebangsaan Malaysia Medical Centre, Kuala Lumpur, MYS; 2 Department of Otolaryngology - Head and Neck Surgery, Hospital Miri, Miri, MYS

**Keywords:** benign inflammatory nasal polyp, nasal obstruction, paranasal sinus, nasal cavity, solitary fibrous tumour

## Abstract

Solitary fibrous tumors of the nasal cavity and paranasal sinuses are rarely encountered in clinical practice. These are unusual mesenchymal tumours initially described as primary spindle-cell neoplasms. Such tumours may manifest in pleural and extrapleural sites such as the liver, parapharyngeal space, sublingual and parotid glands, and thyroid but are seldom described in the nose and paranasal sinus region. Erosion of adjacent structures may occur, but the tumour itself does not metastasise. A young patient presented with a progressive unilateral nasal mass. The initial nasal biopsy reported it as a benign inflammatory nasal polyp. Imaging revealed a large, locally expansile mass within the right nasal cavity displacing the nasal septum. The patient underwent excision of the tumour and the diagnosis of solitary fibrous tumour was confirmed by immunohistochemistry staining. This case is intended to highlight the diagnosis and management of this rare tumour.

## Introduction

A solitary fibrous tumor (SFT) is an unusual tumor previously known as fibrous mesothelioma and described as a primary spindle-cell neoplasm. It was believed that these tumors arise from the mesothelium, but later studies proved that it originates from submesothelial fibroblast-like cells [1]. These are mainly reported in the pleura as well as extrapleural sites such as the liver, parapharyngeal space, sublingual glands, tongue, parotid gland, thyroid, and periorbital region [2]. In rare cases, this tumor arises from the nasal cavity and paranasal sinus with the erosion of adjacent structures [3]. The nose and paranasal sinus involvement in SFT has been sparsely reported in the available literature.

## Case presentation

**Figure 1:**
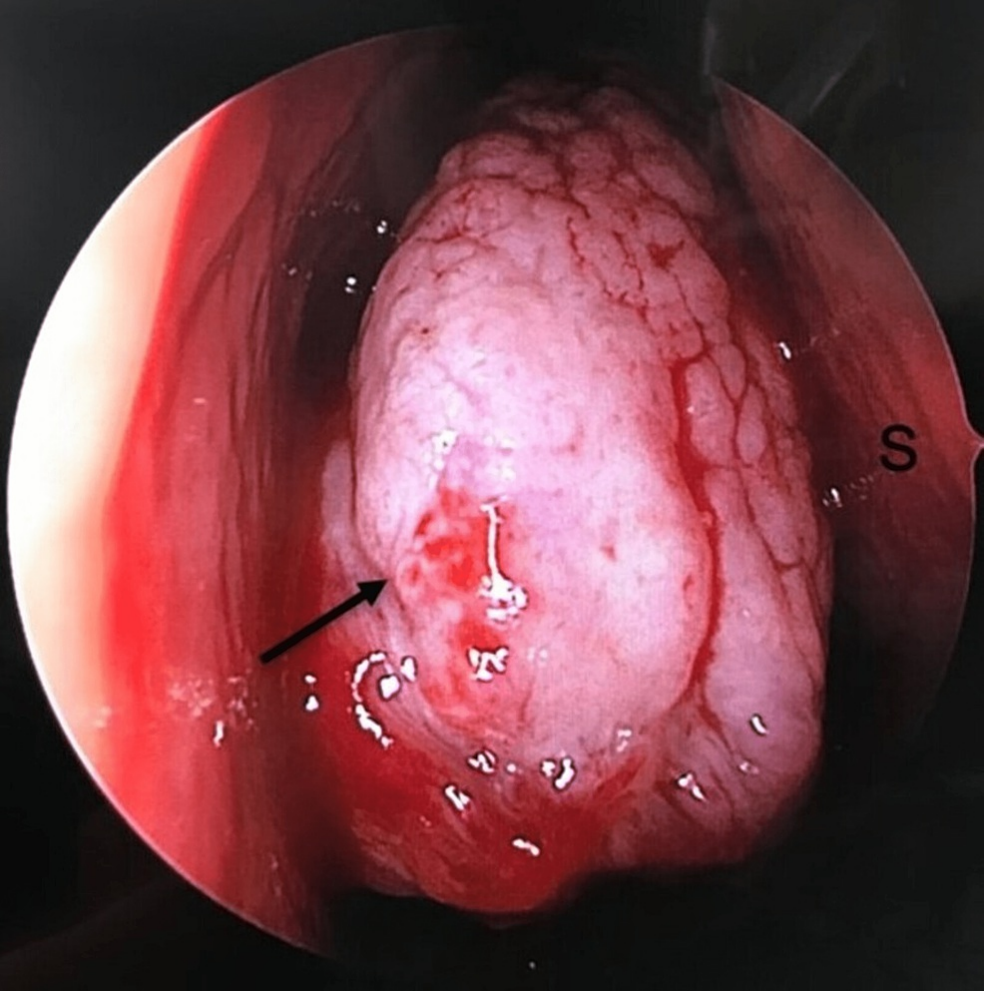
Nasoendoscopy showed a huge solid tumor (arrow) occupying almost the entire right nasal cavity. S: septum

A 24-year-old male presented with progressive right nasal obstruction associated with rhinorrhea for two months. He had no epistaxis, facial pain, or facial swelling. There was no noteworthy past medical or surgical history. Rigid nasoendoscopic evaluation revealed a solid nasal mass with an irregular surface occupying the whole right nasal cavity. The middle turbinate and the osteomeatal complex were obscured by the mass (Figure 1). A biopsy from the nasal mass was obtained.

**Figure 2:**
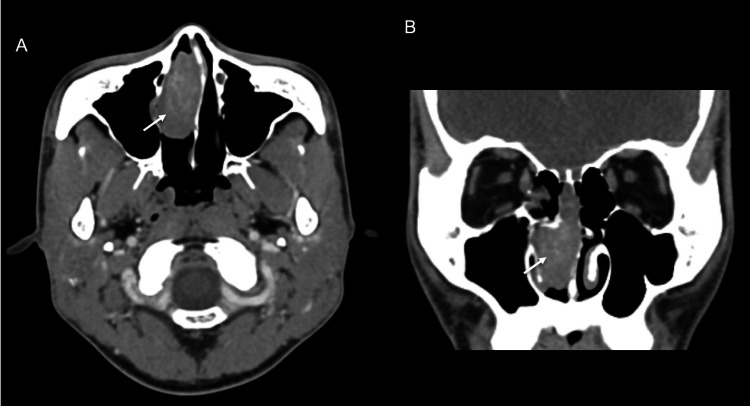
CT depicted a tumor in the right nasal cavity (arrow) causing contralateral bowing of the nasal septum with the erosion of the nasal septum and right lateral nasal wall. (A) Axial cut showed anteroposterior extension of the tumor whereas (B) coronal cut showed superior extension towards the roof of the nasal cavity.

Contrast computed tomography (CT) of the paranasal sinuses showed a well-defined mass, measuring 4.0 cm x 2.1 cm x 3.8 cm, extending superiorly towards the roof of the nasal cavity, displacing the right middle turbinate, obliterating the right osteomeatal complex and causing contralateral bowing of the nasal septum. Slight bony erosion was seen at the middle part of the nasal septum (Figure 2). The paranasal sinuses were clear.

An initial biopsy of the mass during the first visit revealed polypoidal tissue covered by respiratory epithelium, comprising proliferated seromucous glands, and the stroma was infiltrated by inflammatory cells suggestive of a benign inflammatory nasal polyp. Because of the ambiguity of the diagnosis, endoscopic excision of the nasal tumor was done for both diagnostic and therapeutic intention. Intraoperative findings showed a solid mass with an irregular surface and firm consistency, originating from the anterior end of the middle turbinate and occupying the whole right nasal cavity. The adjacent mucosa of the lateral nasal wall and septum were spared. The tumor was removed en bloc using cold instruments. A right middle medial antrostomy was performed and revealed a clear maxillary sinus. No excessive bleeding was observed intra- and postoperatively.

**Figure 3:**
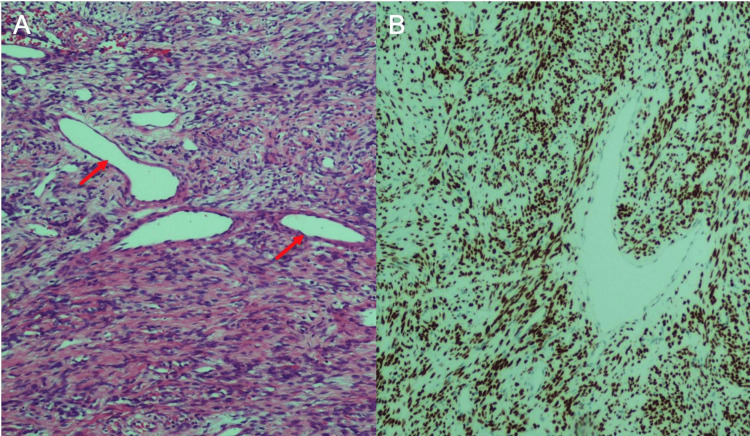
(A) The tumor cells are composed of spindle-shaped cells with varying orientations. Note the ectatic vessels of varying sizes (arrow). (B) The tumor cells are diffusely positive for STAT6 which confirms the diagnosis. STAT6: signal transducer and activator of transcription 6

Pathological examination of the surgical specimen showed a well-circumscribed tumor, the stroma comprising bland spindle-shaped cells proliferation with a focal fascicular pattern. Collagen fibers were seen in between the tumor cells. Mitotic activity was rarely seen. The tumor cells showed focal positivity for CD34 on immunohistochemistry (IHC) staining, with strong and diffuse nuclear staining for signal transducer and activator of transcription 6 (STAT6), which supports the diagnosis of SFT (Figure 3).

**Figure 4:**
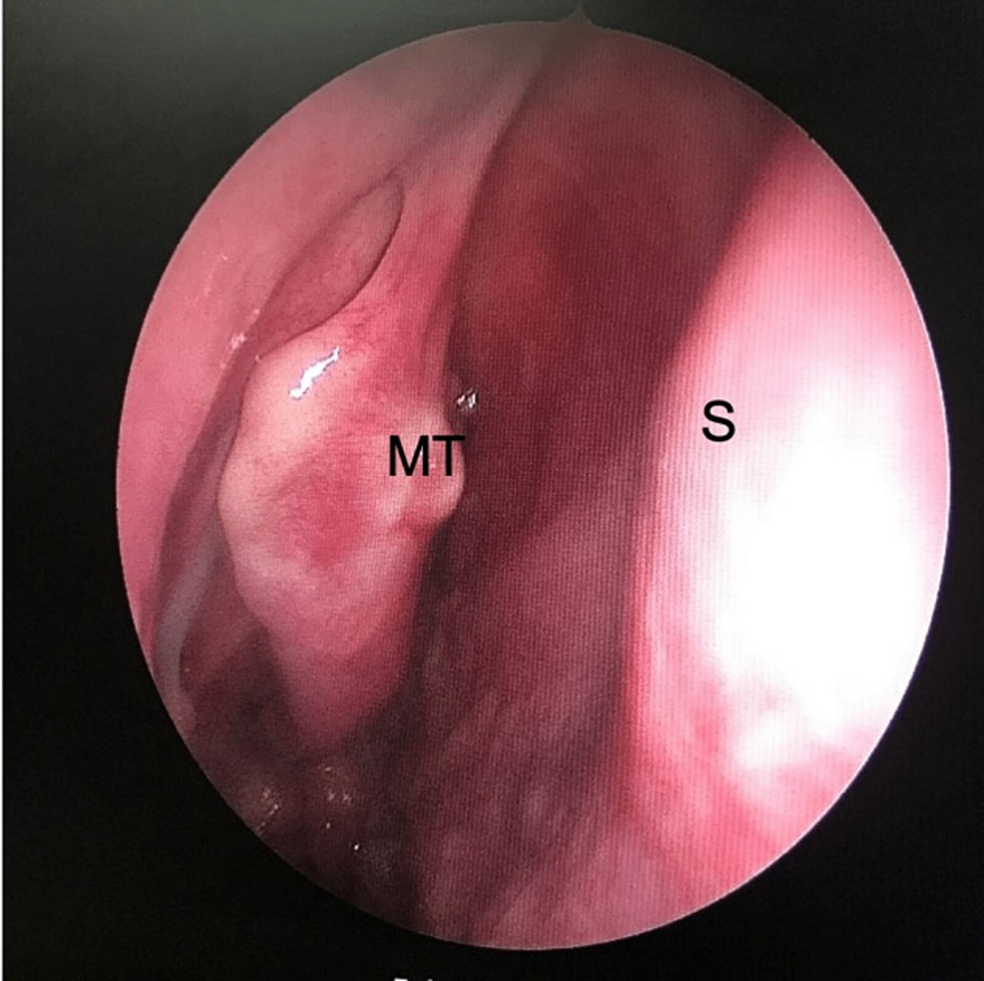
Postoperative nasoendoscopy revealed a spacious right nasal cavity with no signs of recurrence of the tumor. S: septum; MT: middle turbinate

The postoperative recovery was uneventful. At the three-month follow-up, the patient was symptom-free and the nasoendoscopy showed no sign of recurrence (Figure 4).

## Discussion

SFTs are rarely encountered neoplasms. In the past, they were described as fibrous mesothelioma and named primary spindle-cell neoplasms. Klemperer et al. categorized such neoplasms into pleural and extrapleural forms in 1931 [4]. The extrapleural types were rare; in which they can be found in the liver, parapharyngeal space, sublingual glands, tongue, parotid gland, thyroid, and periorbital region [2]. Occasionally, the tumor can arise from the nasal cavity and paranasal sinus area, leading to erosion of adjacent structures [3].

Most extrapleural solitary tumors appear to follow a benign course, presenting as pressure symptoms exerted on nearby structures owing to the progression of the tumor. Similar to the other anatomical sites, there is no gender predilection in nasal cavity SFTs. The presenting age is varied, ranging from 18-79 years with a mean age of 49 years [5]. SFTs, when occurring in the nasal cavity, present with rather non-specific symptoms such as unilateral obstruction, nasal discharge, headache, and sometimes epistaxis. These symptomatologies can accompany any nasal tumor and therefore may not lead to the diagnosis of SFTs. There are a few differential diagnoses of unilateral nasal mass that should be considered including antrochoanal polyps, inverted papilloma, extranasopharyngeal angiofibroma, and malignant tumors such as sinonasal carcinoma and lymphoma.

Endoscopic examination usually reveals these tumors as fibrous and encapsulated masses, filling almost the entire nasal cavity as observed in this case. Histologically, these neoplasms appeared as capsulated tumors made of fibrous tissue and capillaries surrounded by round or spindle cells with vesicular nuclei, without a well-defined growth pattern and with some combinations of different patterns. In IHC staining, these cells are positive for CD34, CD99, and vimentin, whereas negative for keratin, desmin, and S100 protein [6]. It is worth mentioning that in our case, the initial biopsy that was performed in the clinic preoperatively was reported as an inflammatory polyp. This emphasizes the importance of the IHC study in establishing an accurate diagnosis.

CT is so far the best imaging modality for diagnosis. This enables the assessment of tumor extension and the amount of bone involvement. CT findings show the tumor homogeneously isoattenuating to gray matter. It can be complemented by magnetic resonance imaging (MRI), especially in cases with orbit or intracranial extension [7,8].

The treatment of choice is endoscopic surgical resection. It is a minimally invasive procedure and allows complete clearance. In SFTs not amenable to en bloc resection, piecemeal resection with subsequent elevation and resection of the underlying periosteum can be performed to minimize recurrence. Given the small number of SFTs and limited understanding of these tumors, a limited number of treatment results are available in the literature. Some other treatment options were also practiced in the past. Rizzo et al. performed preoperative embolization in their patient to reduce hemorrhage because the tumor appeared reddish endoscopically, but other authors with a different school of thought have proceeded with treatment without prior embolization procedure and reported no intraoperative bleeding complications [9]. Martinez et al. called for radiotherapy after the removal of nasal cavity SFTs to minimize the risk of recurrence. Having said that, the use of radiotherapy in these benign tumors remains controversial and there is currently insufficient evidence to recommend their use. The reported recurrence rate for SFTs varied from 5-40% [10,11]. The overall prognosis of SFTs is considered favorable after complete resection. However, due to the limited number of SFTs in the nasal cavity, the accurate prediction of their clinical behavior and prognosis is difficult.

## Conclusions

Although uncommon, SFTs can occur in the nasal cavity and should be considered in the array of differential diagnoses of unilateral nasal mass. Complete surgical excision remains the mainstay of treatment for SFTs. SFTs that are not amenable to en bloc resection can be removed piecemeal followed by resection of the underlying periosteum is recommended. Recurrence is uncommon following complete resection. 
